# Supplementary material for: SIRT7 as a context-dependent biomarker and therapeutic target: Insights from a pan-cancer study
Source: PLoS One. 2026 Feb 5;21(2):e0342269. doi: 10.1371/journal.pone.0342269 (PMC12875470; doi:10.1371/journal.pone.0342269)
Supplement: S2 Fig — (A) K-Means clustering, (B) MCL Clustering, and (C) DBSCAN clustering. (DOCX) [file pone.0342269.s002.docx]

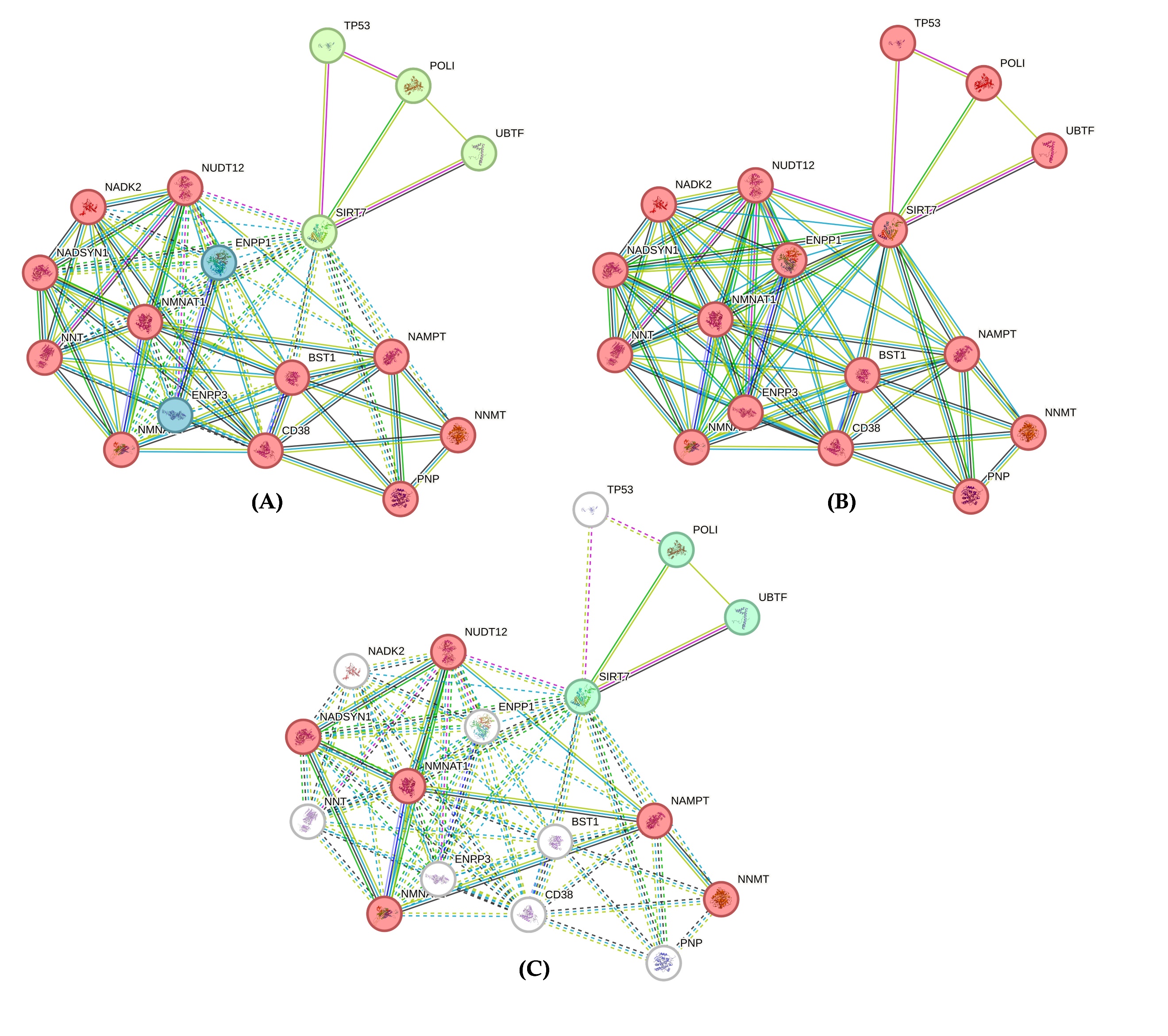


**Supplementary Figure S2.** Identification of protein clusters. (A) K-Means clustering, (B) MCL Clustering, and (C) DBSCAN clustering (Source: STRING 12).
