# Supplementary material for: SIRT7 as a context-dependent biomarker and therapeutic target: Insights from a pan-cancer study
Source: PLoS One. 2026 Feb 5;21(2):e0342269. doi: 10.1371/journal.pone.0342269 (PMC12875470; doi:10.1371/journal.pone.0342269)
Supplement: S3 Fig — (A) Deep learning neural network model. (B) Internal validation R2 over 1000 epochs, (C) Loss of training and validation sets over 1000 epochs (generated using matplotlib 3.10.0 of Python 3.12.11). (DOCX) [file pone.0342269.s003.docx]

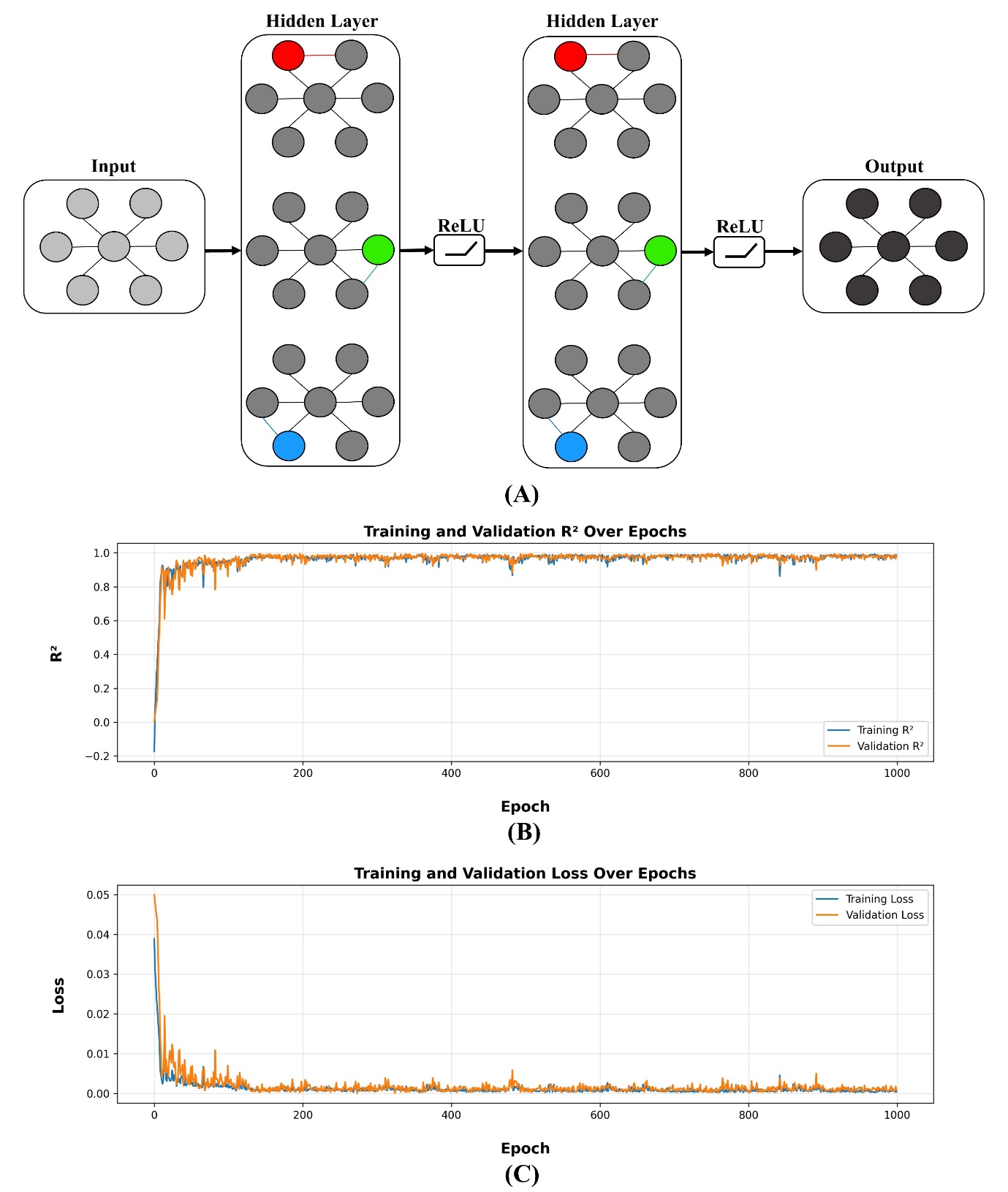


**Supplementary Figure S3.** Automated deep learning model for 12-centrality metrics calculations. (A) Deep learning neural network model. (B) Internal validation R^2^ over 1000 epochs, (C) Loss of training and validation sets over 1000 epochs (generated using matplotlib 3.10.0 of Python 3.12.11).
