# Supplementary material for: SIRT7 as a context-dependent biomarker and therapeutic target: Insights from a pan-cancer study
Source: PLoS One. 2026 Feb 5;21(2):e0342269. doi: 10.1371/journal.pone.0342269 (PMC12875470; doi:10.1371/journal.pone.0342269)
Supplement: S4 Fig — (A) The most important features for the deep learning model. (B) Statistical analysis of 12 centrality metrics for proteins. (DOCX) [file pone.0342269.s004.docx]

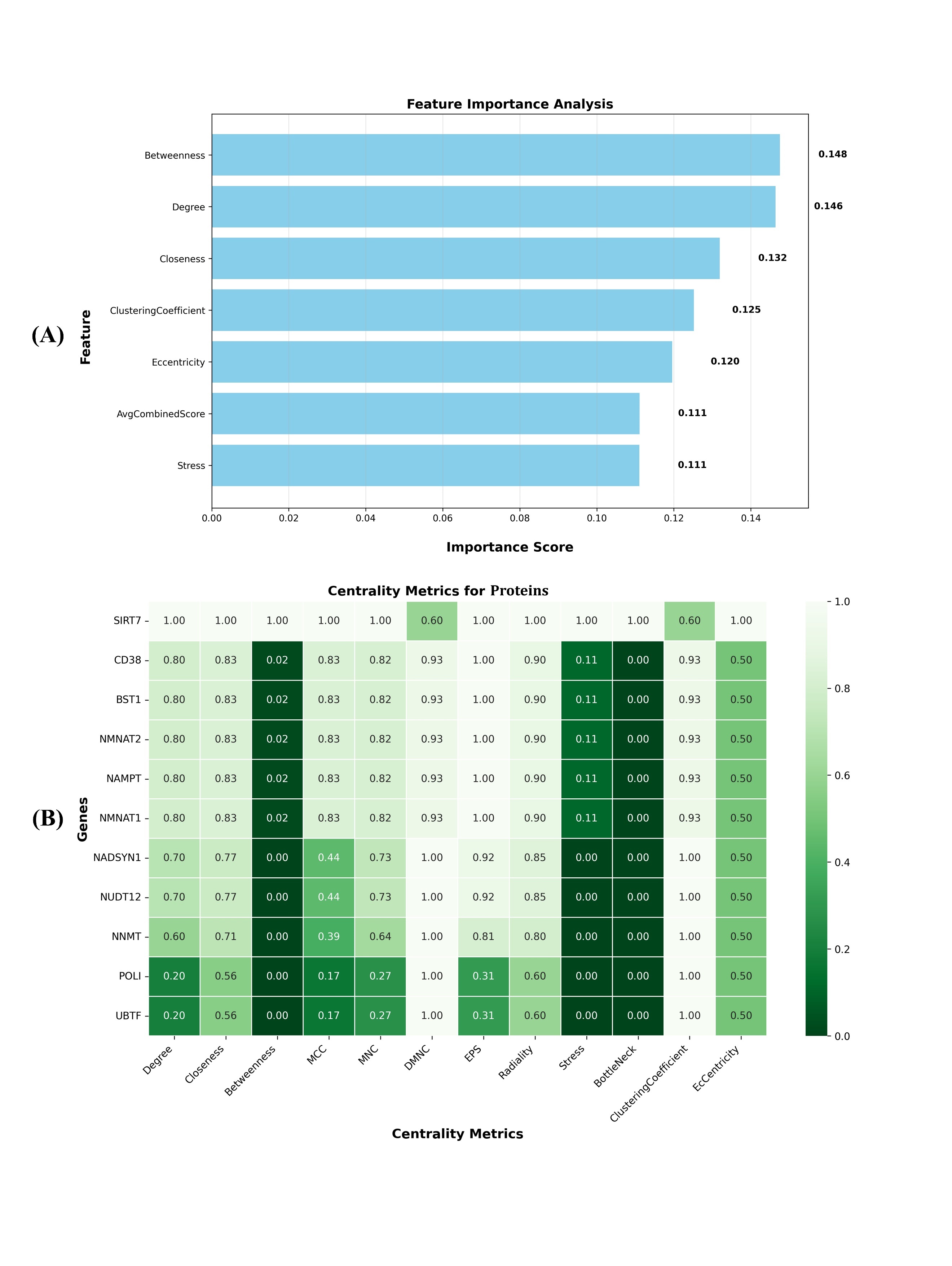


**Supplementary Figure S4.** Influence of Centrality Metrics. (A) The most important features for the deep learning model. (B) Statistical analysis of 12 centrality metrics for proteins (generated using matplotlib 3.10.0 of Python 3.12.11).
