# Supplementary material for: SIRT7 as a context-dependent biomarker and therapeutic target: Insights from a pan-cancer study
Source: PLoS One. 2026 Feb 5;21(2):e0342269. doi: 10.1371/journal.pone.0342269 (PMC12875470; doi:10.1371/journal.pone.0342269)
Supplement: S5 Fig — (A) Dot plot for GO enrichment, and (B) Bar plot for pathway enrichment analysis (generated using matplotlib 3.10.0 of Python 3.12.11). (DOCX) [file pone.0342269.s005.docx]

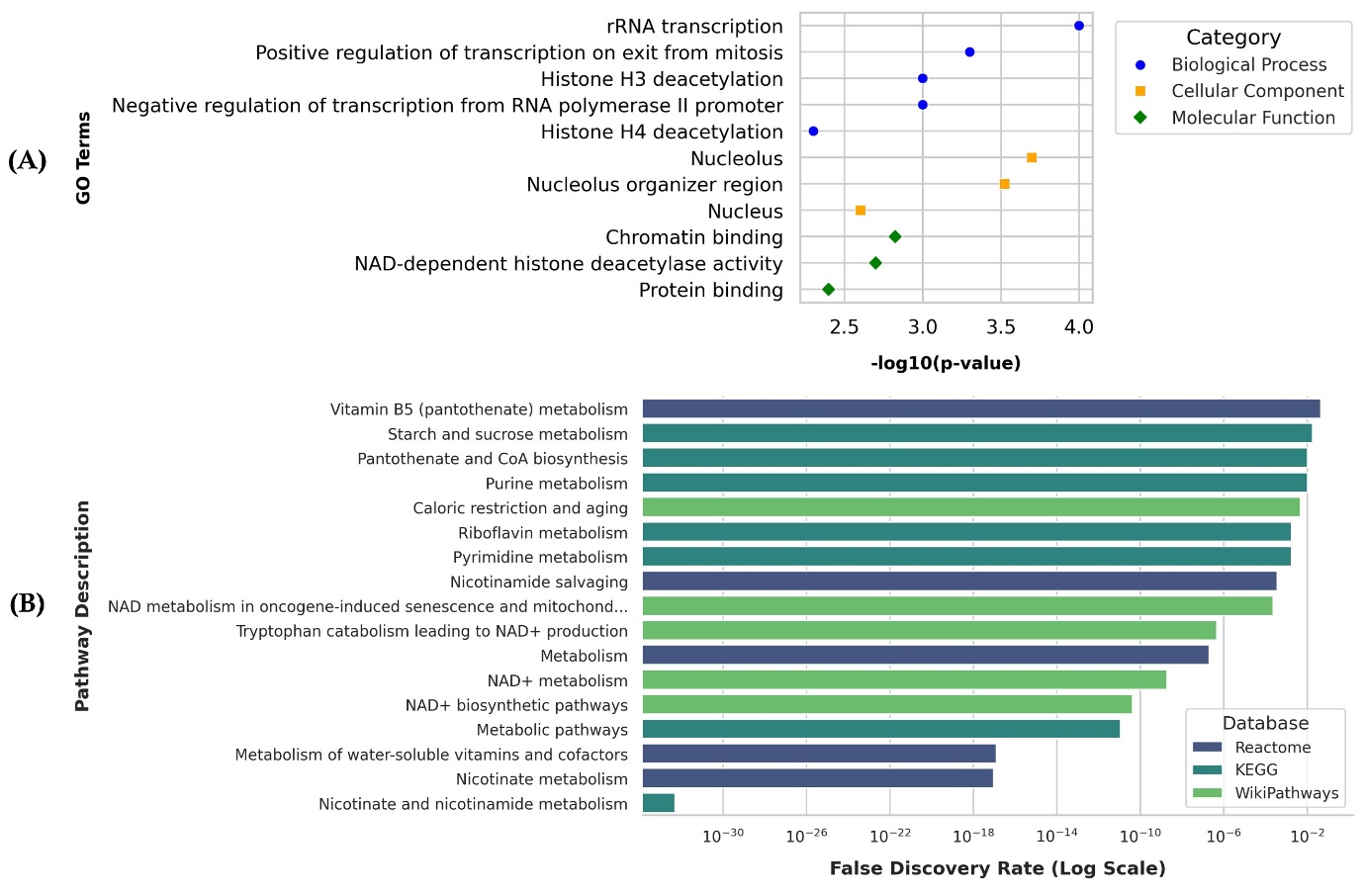


**Supplementary Figure S5.** GO and Pathway enrichment analysis. (A) Dot plot for GO enrichment, and (B) Bar plot for pathway enrichment analysis (generated using matplotlib 3.10.0 of Python 3.12.11).
