# Supplementary material for: SIRT7 as a context-dependent biomarker and therapeutic target: Insights from a pan-cancer study
Source: PLoS One. 2026 Feb 5;21(2):e0342269. doi: 10.1371/journal.pone.0342269 (PMC12875470; doi:10.1371/journal.pone.0342269)
Supplement: S6 Fig — (DOCX) [file pone.0342269.s006.docx]

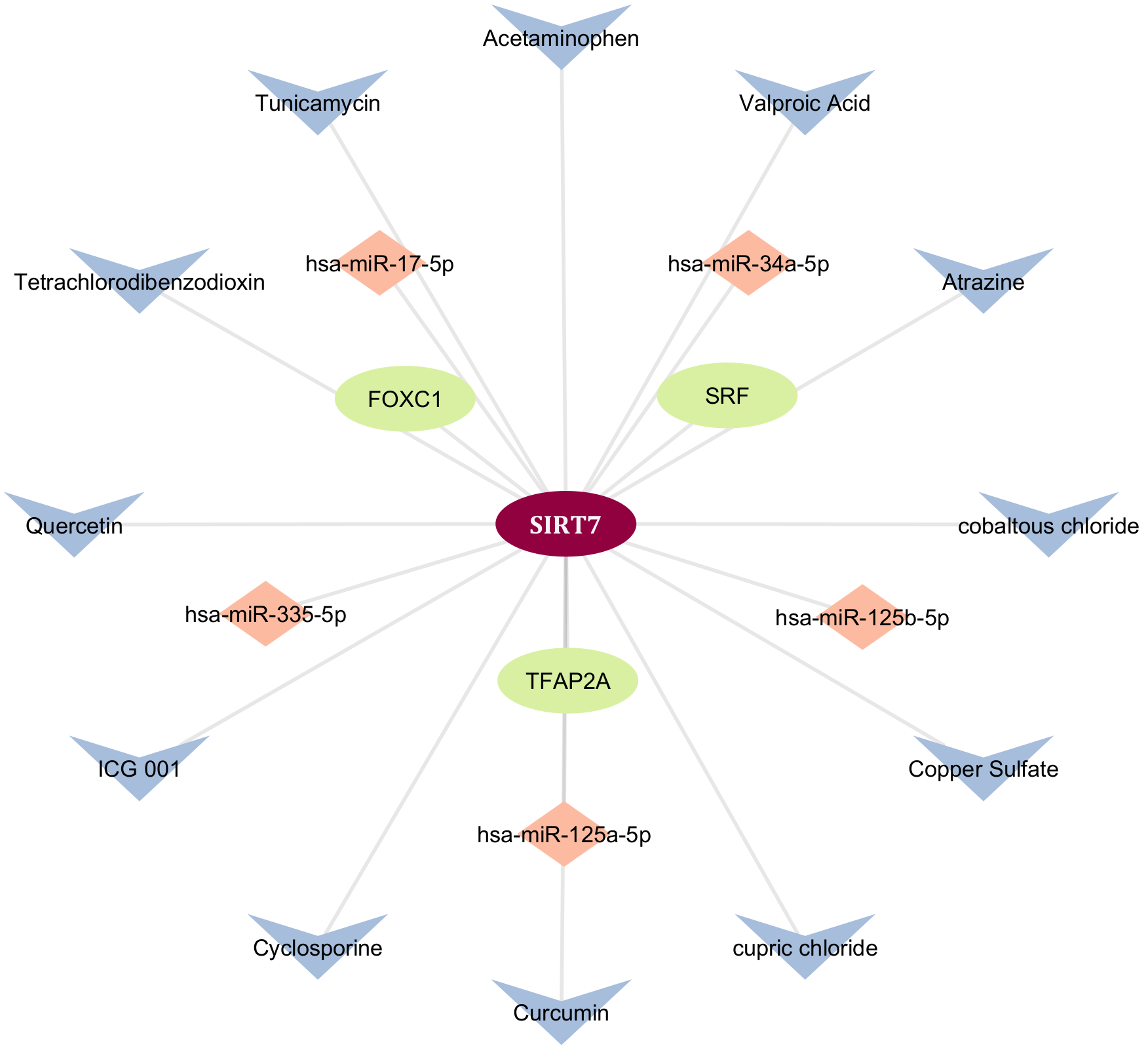


**Supplementary Figure S6. Gene regulatory network illustrating interactions among SIRT7, transcription factors (Green), microRNAs (Red), and modulatory compounds (Blue) (Cytoscape 3.10.3).**
