# Supplementary material for: SIRT7 as a context-dependent biomarker and therapeutic target: Insights from a pan-cancer study
Source: PLoS One. 2026 Feb 5;21(2):e0342269. doi: 10.1371/journal.pone.0342269 (PMC12875470; doi:10.1371/journal.pone.0342269)
Supplement: S2 Table — (DOCX) [file pone.0342269.s008.docx]

**Supplementary Table S2.** Detailed pharmacophore features with spatial coordinates and activation status.

| **Feature** | **Status** | **X** | **Y** | **Z** | **Radius (r)** |
| --- | --- | --- | --- | --- | --- |
| Aromatic | ON | 0.4 | -2.9 | 2.2 | 1.0 |
| Aromatic | ON | 4.1 | 3.7 | 0.9 | 1.0 |
| NegativeIon | OFF | 1.6 | 1.2 | 1.4 | 1.0 |
| HydrogenDonor | ON | 8.5 | 6.2 | 1.2 | 1.0 |
| HydrogenDonor | ON | 2.7 | 6.3 | 1.5 | 1.0 |
| HydrogenAcceptor | ON | 10.1 | 6.4 | 5.1 | 1.0 |
| HydrogenAcceptor | ON | 8.4 | 4.9 | -0.5 | 1.0 |
