# Supplementary material for: SIRT7 as a context-dependent biomarker and therapeutic target: Insights from a pan-cancer study
Source: PLoS One. 2026 Feb 5;21(2):e0342269. doi: 10.1371/journal.pone.0342269 (PMC12875470; doi:10.1371/journal.pone.0342269)
Supplement: S3 Table — (DOCX) [file pone.0342269.s009.docx]

**Supplementary Table S3.** Screening of Compounds from the publicly available databases based on pharmacophore features.

| **Database** | **Total Compounds** | **Identified Compounds** |
| --- | --- | --- |
| ChEMBL | 2,264,112 | 30 |
| ChemSpace | 7,659,928 | 1 |
| CMNPD | 47,451 | 2 |
| IMPPAT | 17,792 | 5 |
| MCULE | 39,843,637 | 4 |
| MCULE Ultimate | 126,471,502 | 1 |
| MolePort | 4,742,020 | 1 |
| ZINC | 13,127,550 | 8 |
