# Supplementary material for: SIRT7 as a context-dependent biomarker and therapeutic target: Insights from a pan-cancer study
Source: PLoS One. 2026 Feb 5;21(2):e0342269. doi: 10.1371/journal.pone.0342269 (PMC12875470; doi:10.1371/journal.pone.0342269)
Supplement: Attachment — Submitted filename: SIRT7 response.docx [file pone.0342269.s012.docx]

05 November, 2025

Firoz Ahmed
Academic Editor
PLOS ONE

Dear Editor,

Special gratitude to you for allowing us to resubmit a more revised and updated version of our manuscript entitled **"SIRT7 as a Context-Dependent Biomarker and Therapeutic Target: Insights from a Pan-Cancer Study" (Manuscript ID: PONE-D-25-42611)** to the PLOS ONE journal as an original research article. We have organized the manuscript based on the editor’s and reviewers’ comments. In response to valuable suggestions, we have modified, revised, organized, corrected, and narrowed the manuscripts. The result section is now presented with a clear rationale for each step, and a logical flow has been maintained. Some figures have now been presented as supplementary figures to make the manuscript more concise. However, due to it being a pan-cancer study, we tried to provide logical and relevant information as much as possible, and all the limitations have been addressed in the discussion section. We also provided some logical explanations of particular queries of the honorable reviewers and editor, particularly regarding the use of the AlphaFold3 modeled structure rather than the crystal structure. Additionally, we have appropriately addressed all queries of reviewers in the response letter, line by line. We hope our response below will successfully address the reviewer’s specific points and improve the manuscript. Please let us know if you have any additional questions or comments. Finally, we would like to express our thanks and gratitude for your kind cooperation.

Sincerely yours,

Shahin Mahmud

Assistant Professor

Department of Biotechnology and Genetic Engineering

Mawlana Bhashani Science and Technology University, Santosh, Tangail-1902, Bangladesh.

Email: [shahin018mbstu@gmail.com](mailto:shahin018mbstu@gmail.com)

**Responses to the honorable reviewers’ s comments**

**Additional Editor Comments:**

Dear Dr. Mahmud,

Thank you for submitting your manuscript entitled “SIRT7 as a Context-Dependent Biomarker and Therapeutic Target: Insights from a Pan-Cancer Study.” The reviewers have now completed their evaluation, and I am attaching their comments. Please review these carefully and address each point in detail.
In addition to the reviewers’ feedback, I have the following editorial suggestions. These need to be implemented to strengthen and improve the quality of the manuscript:

| **SL.** | **Comments/Questions/Instruction/Guidelines** | **Responses of the Authors** |
| --- | --- | --- |
| 1. | Although the manuscript uses multiple techniques, there is insufficient rationale and justification for their selection, and in many places the analyses are not logically connected. The manuscript is large and needs to be more focused—clearly defining the core problem and the steps to address it. | We have now clearly explained and justified the rationale for the selection of each technique and connected them to focus on justifying the title of our manuscript.  Specifically, we have now focused on   (1) Determining the biomarker possibility of SIRT7 by network, expression, survival, alteration, and immune association analysis. (2) exploring therapeutic opportunities by identifying active sites, inhibitory sites, molecular docking, molecular dynamics, and post-dynamics MMPBSA studies.   To justify our study as a pan-cancer study, we have to use several techniques, which we have clarified now. Therefore, the manuscript is large. However, we have trimmed our manuscript by focusing on the core problem and keeping the steps sequential that are required to address the problem. Minor or less important techniques have been merged or excluded, and some repetitive or less important figures are presented now as supplementary figures.  We have designed this study in a way that researchers who are interested in SIRT7 may find valuable information and insights for subsequent experiments. |
| 2. | Furthermore, the crystal structure of SIRT7 (or of at least parts of it) is already available. Given these existing structural data, the authors must explain why they chose to use AlphaFold to generate the SIRT7 structure in their work. | Thank you for raising this issue. Although experimental structures are available, we did not find them suitable for our study. Before starting our study, we have thoroughly reviewed the structure of SIRT7 to identify any crystal structure of SIRT7 with co-crystallized ligand binding at the inhibitory site. However, no complete crystal structure of SIRT7 is available in the PDB database. Though some fragments are available, they are not suitable for subsequent molecular docking analysis due to the lack of a proper inhibitory and catalytic site.  Additionally, the mentioned structure, 9GMK, has been resolved using Electron Microscopy and does not provide any information about inhibitory sites. Therefore, we have queried the literature to identify the inhibitory site information and presented it in **Figure 11**.  We have performed molecular docking targeting both the Electron Microscopic structure and the modeled structure with the experimentally validated SIRT7 inhibitor (PubChem CID: 155513088).  In terms of docking, the AlphaFold3 modeled structure suppressed the EM structure that is available at legacy PDB format: 9GMK; however, the resolution is lower (3.5 Å), and that was reflected in docking studies, where we have observed a lower binding affinity in the case of the EM structure than the AlphaFold3 model. Most interestingly, the inhibitors bind perfectly to the modeled structure at both the literature-based identified inhibitory sites and active sites (**Figure 11, Table 6,** and **Figure 13**). This specific binding and interaction pattern was possible due to the development of our pharmacophore model, which was developed based on amino acid residues of both inhibitory sites and catalytic sites of the main pocket.  Earlier, we stated the unavailability of a crystal structure. Although some fragments of the crystal structure are available, they are not suitable to perform docking. The resolution of the EM structure is also not high (3.5Å) and exhibited much inferior performance than our modeled structure in terms of binding affinity. Therefore, the Alphaold3 model of SIRT7 structure has been used in this study. However, we hope our study will instigate proteomics researchers to resolve the complete crystal structure of SIRT7.  We have mentioned the limitations of the modeled structure and recommend resolving the complete crystal structure of SIRT7 in the discussion section.  Thank you for guiding us to improve our manuscript. |

**Honorable Reviewer 1:**

Major Comments

| **SL.** | **Comments/Questions/Instruction/Guidelines** | **Responses of the Authors** |
| --- | --- | --- |
| 1. | 1. Overuse of Computational Methods Without Clear Rationale  The authors employ numerous techniques—machine learning, molecular docking, pharmacophore modeling, survival analysis, network analysis, and more—without a coherent rationale or integration. This excessive use of methods appears unfocused and gives the impression of trying to include as much data as possible rather than answering a specific biological question. The manuscript would benefit from narrowing its scope and focusing on a few well-justified approaches. | Thank you for addressing this issue. The reason behind choosing several techniques is that it is a pan-cancer study, which required multidimensional analysis to strengthen and support findings.  We have reviewed the entire manuscript and have now justified the rationale for integrating these techniques at each step. However, we have narrowed our scope by excluding minor, less impactful, or repetitive techniques as per your suggestions.  In the revised manuscript, we have particularly focused on justifying the biomarker and therapeutic opportunities, but still tried to provide most of the relevant information with a clear rationale to justify our pan-cancer study.  We have primarily divided our study into 3 parts:  1. Network analysis to determine how important SIRT7 is in the biological network of SIRT7 partners. We found SIRT7 is the central hub among their partners.  2. Determines the Context-dependent role of SIRT7 in cancer using gene expression, survival, mutational, and immune association studies, where we have found that the overexpression of SIRT7 is responsible for several cancers, supported by our literature review study (**Table 3**). However, in some cancer types, underexpression is also related to cancer progression, such as the underexpression of SIRT7 is responsible for the progression of breast cancer [1,2].  3. Identification of novel inhibitors using computational approaches, where we have identified 3 promising compounds. |
| 2. | 2. Lack of Solid Biological Findings  Despite the extensive data analysis, the manuscript does not present any strong or novel biological insights. | Thank you for addressing this query. Our study aims to present the context-dependent role of SIRT7 in different types of cancers, as we found that the role of SIRT7 is not straightforward in cancer. In some cancer types, the overexpression of SIRT7 has been observed, and in some cases, underexpression. We have found that the overexpression of SIRT7 is better for the survival of sarcoma, and because this is a novel finding of our study therefore we have recommended the requirement of further experimental validation to confirm these findings. Moreover, only a single previous study presents that the overexpression of SIRT7 is related to a poor outcome for the survival of osteosarcoma patients. However, no specific studies have been conducted for soft tissue sarcoma, and therefore, due to this gap in the currently available literature, the requirement for more rigorous studies has been suggested in our study.  Besides, we have identified 3 promising novel inhibitors using computational approaches: ZINC000150487575, ZINC000150641215, and MCULE-8582664468. |
|  | The results are largely descriptive, and there is minimal effort to connect computational outputs to known or hypothesized biological mechanisms involving SIRT7. For example, enriched GO terms such as rRNA processing are mentioned but not explained in the context of SIRT7’s established role in nucleolar function and ribosome biogenesis. | We have now revised our manuscript to show how computational findings can be connected to known biological mechanisms, particularly in the case of GO and pathway enrichment analysis. We have addressed and highlighted this in the discussion section of our manuscript.  SIRT7 exhibits critical regulatory functions in chromatin remodeling and transcriptional modulation, and is predominantly localized within nuclear and nucleolar compartments, as indicated by the identified GO terms. This enzyme maintains nucleolar structural integrity and facilitates ribosomal RNA transcription [3]. Through its catalytic activity on histones H3 and H4, SIRT7 orchestrates transcriptional programs essential for cell cycle progression. The enzyme's requisite NAD⁺ dependence establishes a fundamental connection between cellular bioenergetics and epigenetic regulation [4,5], highlighting its significance in nucleolar homeostasis and metabolic reprogramming in oncogenic contexts [6]. |
| 3. | 3. No Experimental Validation  The manuscript proposes SIRT7 as a therapeutic target but does not include any experimental data to support this claim. Validation in biological systems (e.g., cell lines, patient samples) is essential to substantiate computational findings and enhance translational relevance. | In this case, we have designed our study based on the below:   1. We have chosen a reference potent inhibitor of SIRT7 from previously experimentally validated evidence. 2. Then, we have compared this reference inhibitor throughout our study to provide comparative insights on SIRT7 inhibitory capability.   Due to current resource and budget limitations, we are unable to perform further experimental validation. However, because our findings are promising, supported by both molecular docking and dynamics simulation studies, the dissemination of our findings will inspire other researchers to perform experimental studies who have no resources or budget limitations. |
| 4. | 4. Unsubstantiated Context-Dependent Role  Although the title emphasizes SIRT7’s context-dependent behavior, the manuscript does not provide sufficient comparative analysis or mechanistic explanation to support this claim. The context-dependence remains an untested assertion. | Thank you for addressing this issue. In the revised manuscript, we have particularly addressed why we have stated a context-dependent role.  We have provided a detailed table summarizing the specific mechanisms by which SIRT7 is involved in cancer progression, based on our literature review analysis of recently published literature (**Table 3**). This analysis supports our subsequent study of exploring novel inhibitors for SIRT7.  Additionally, our computational analysis suggests that SIRT7 expression is not straightforward; rather, it is overexpressed in certain cancer types and underexpressed in certain cancer types, which provides insight into the context-dependent role of SIRT7. These computational findings are also supported by the recent literature, and some contradictory findings are also observed. Moreover, previous studies identified that the underexpression of SIRT7 is responsible for the progression of breast cancer [1,2].  This observation reinforces the requirement of this pan-cancer study to clarify the context-dependent role of SIRT7, where both similar and contradictory findings have been observed. Here, we have performed the gene expression analysis, survival analysis, mutation impact, and immune association analysis to address that the role of SIRT7 is context-dependent and its expression can vary in cancer types and can be modulated by mutational impact, CD8+ immune cell impact.  Hence, our study could guide researchers interested in SIRT7’s involvement in diverse cancer types.  However, indeed, we did not particularly focus on mechanistic insight. It is possible to conduct a standalone mechanistic study on SIRT7. In this pan-cancer study, we focus on broad aspects of cancer. We have now clarified and highlighted the context-dependent issue more precisely in the results and discussion section of the manuscript. |
| 5. | Please improve the quality and resolution of discussed figures. | Thank you for your suggestion. All figures in the manuscript have now been provided with improved quality and higher resolution (>300 dpi). |

**Minor Comments**

| **SL.** | **Comments/Questions/Instruction/Guidelines** | **Responses of the Authors** |
| --- | --- | --- |
| 1. | • Poor Figure Resolution Across the Manuscript  All figures suffer from low resolution and poor formatting, making them difficult to read and interpret. Text is often blurry or too small, and visual elements lack clarity. The authors should regenerate all figures at high resolution, with appropriate font sizes, labeling, and formatting suitable for publication. | We have thoroughly checked all the figures to identify figures with low quality and resolution. We have now ensured all figures in the manuscript with improved quality and higher resolution, which is suitable for publication. |
| 2. | • Improper Attribution in Figure 3b  Figure 3b appears to include an image from the Human Protein Atlas, but this is not acknowledged in the figure legend or main text. The authors must properly cite the source and ensure compliance with licensing terms. | We have read the citation and license agreement of Human Protein Atlas and fulfill the criteria to use the resources from HPA by citing one primary source and all secondary sources. |
| 3. | • Language and Style  The manuscript would benefit from thorough language editing to improve grammar, flow, and scientific tone. | The manuscript has been thoroughly reviewed, where grammar, flow, and scientific tone have been substantially improved. |
| 4. | • Discussion of Limitations  The authors should explicitly acknowledge the limitations of relying solely on in silico analyses and public datasets, including potential biases and lack of experimental control. | We have clearly discussed the limitations of in silico analyses more rigorously and recommended future experimental suggestions. |
| We sincerely appreciate the time and effort you invested in reviewing our manuscript. Your detailed and constructive feedback guided important revisions that substantially improved the clarity, rigor, and overall quality of our work. Thank you for your thoughtful and insightful suggestions. | | |

**Honorable Reviewer 2:**

**I have a few minor concerns as outlined below:**

| **SL.** | **Comments/Questions/Instruction/Guidelines** | **Responses of the Authors** |
| --- | --- | --- |
| 1. | The crystal structure of SIRT7 is already available (e.g., PDB ID: 9GMK), albeit in complex with DNA and histones. Why was there a need to model the full protein instead of utilizing or modifying the available structure? | Thank you for this important concern. We were seeking a suitable crystal structure of SIRT7 with a co-crystallized inhibitor that could guide us to identify the inhibitory sites for subsequent molecular docking analysis. Unfortunately, we were unable to find a complete crystal structure of SIRT7. The mentioned structure has been resolved using Electron Microscopy and does not provide any information about inhibitory sites. Therefore, this is the only reason we have queried the literature to identify the inhibitory site information and presented it in **Figure 11**.  We have performed molecular docking targeting both the Electron Microscopic structure and the modeled structure with the experimentally validated SIRT7 inhibitor (PubChem CID: 155513088).  In terms of docking, the AlphaFold3 modeled structure suppressed the EM structure that is available at legacy PDB format: 9GMK; however, the resolution is lower (3.5 Å), and that was reflected in docking studies, where we have observed a lower binding affinity in the case of the EM structure than the AlphaFold3 model. Most interestingly, the inhibitors bind perfectly to the modeled structure at both the literature-based identified inhibitory sites and active sites (**Figure 11, Table 6,** and **Figure 13**). This specific binding and interaction pattern was possible due to the development of our pharmacophore model, which was developed based on amino acid residues of both inhibitory sites and catalytic sites of the main pocket.  Earlier, we stated the unavailability of a crystal structure. Although some fragments of the crystal structure are available, they are not suitable to perform docking. The resolution of the EM structure is also not high (3.5Å) and exhibited much inferior performance than our modeled structure in terms of binding affinity. Therefore, the Alphaold3 model of SIRT7 structure has been used in this study. However, we hope our study will instigate proteomics researchers to resolve the complete crystal structure of SIRT7.  We have mentioned the limitations of the modeled structure and recommend resolving the complete crystal structure of SIRT7 in the discussion section. |
| 2. | While identifying the active site residues, which specific function of the protein are you considering? How would inhibition of that function contribute to cancer management or therapy? | We have performed a thorough literature review to collect information on inhibitory sites and active sites. However, data is very scarce, and we have found only one clear inhibitory site information from literature, and also some studies have addressed that catalytic sites are responsible for the inhibition mechanism. Moreover, based on previous studies, SIRT7 overexpression is linked to diverse cancer types (**Table 3**). Therefore, we have considered an inhibitory strategy by identifying inhibitory sites for cancer management or therapy. |
| 3. | In Figure 15, you mention inhibitory and catalytic residues; however, only a single reference (Reference 80, for inhibitory residues) is cited, and without sufficient detail. Please provide more information from this reference and, if available, cite additional literature supporting the identified active site residues. | We have already acknowledged that the inhibitory site information for SIRT7 is very scarce. Only a single study that we have cited provided specific inhibitory site information. However, we have incorporated available literature that addresses the active site information. Initially, we have collected active site information from the NCBI database, which collects information from available literature.  The selection of our reference SIRT7 inhibitor is also from this reference 80 (currently 90), which we have discussed and compared throughout the study. |
| 4. | All compounds selected for molecular docking have molecular weights greater than 500, thereby violating the first criterion of Lipinski’s Rule of Five. How do you justify the druggability of these compounds? | Thank you for your valuable query. In the case of being cancer drug, following Lipinski’s Rule is not straightforward. A lot of drugs that don’t align with none of the criteria of Lipinski’s Rule have indeed been approved by both FDA and US as cancer drugs, such as Everolimus (958.2 g/mol), which is an mTOR-PI3K pathway inhibitor, Venetoxlax (868.4 g/mol), a BCL2 inhibitor, Paclitaxel (853.9 g/mol) used to treat cancer of the lungs, ovaries, and breasts are just a few examples.  However, for small molecule identification from natural sources, we also think Lipinski’s Rule should be followed so that after any modification to the structure, the structure should still remain in a non-toxic and drugable state. |
| 5. | The toxicity analysis appears incomplete. Please expand it to include other key parameters. In addition, provide a complete tabulation of Lipinski’s Rule of Five compliance for the selected compounds. | Thank you for this important observation. We have already provided the most important toxicity information (hepatotoxic and carcinogenicity) in **Table 7** using the ProTox 3.0 server. However, we have noticed that we did not mention ProTox in the method section. This is our accidental oversight. We have now provided toxicity profiling with probability scores.  However, we have already clarified that in the case of cancer drugs, or even in the case of prominent antibiotics, Lipinski’s Rule of Five has not been considered as a selection criterion in many cases. |
| 6. | Are there any previous reports of small molecules or chemical compounds being identified as inhibitors of SIRT7? If so, please cite and discuss them. | Yes, we have utilized the reference of our study, a potent inhibitor of SIRT7 [7]. We designed our study based on this experimentally validated inhibitor and compared and discussed it for molecular docking and dynamics simulation. We have already cited the reference (previously 80, currently 90). Thank you for your important suggestion. |
| 7. | Kindly overlay each of the selected docking compounds onto the pharmacophore model individually to illustrate their fit. | Thank you for this interesting suggestion. We have now presented it in **Figure 12 (B-D)** as per your suggestion. |
| 8. | Please remove retracted articles from the reference list (e.g., Reference No. 88) and strengthen the manuscript by citing more recent and relevant publications. | Thank you for this critical observation. We have now retracted ref 88 and cited more recent and relevant publications to justify our statement. |
| 9. | You mention that high SIRT7 expression correlates with poor survival, particularly in sarcoma. What do previous reports state on this correlation, and how consistent are your findings with existing literature? | Only a single previous study has stated that high SIRT7 expression correlates with poor prognosis, particularly in osteosarcoma patients, and contrasts with our findings [8].  However, this is in the case of bone sarcoma; in the case of soft tissue sarcoma, such as liposarcoma or clear cell sarcoma, no studies have been found.  Therefore, we have now mentioned and highlighted the requirements of further investigation to validate our findings in the discussion section of our manuscript. |
| We sincerely thank you for the time and expertise you devoted to reviewing our manuscript. Your thorough and constructive feedback was invaluable and helped us address key issues, substantially improving the clarity, coherence, and overall quality of our work. | | |

**Editor’s Comments on the Manuscript**

| **SL.** | **Comments/Questions/Instruction/Guidelines** | **Responses of the Authors** |
| --- | --- | --- |
| 1. | Logical Flow & Conciseness  As one reviewer pointed out, the Results section appears disjointed and lacks logical continuity. The manuscript should be reorganized so that each analysis builds on the previous one in a clear scientific progression. Additionally, the manuscript is quite long; please trim unnecessary parts and present results more concisely. | Thank you for your valuable suggestions. We have thoroughly reviewed our manuscript, and any unnecessary or repetitive techniques have been excluded, merged, or presented in the supplementary section of the manuscript to make the manuscript more concise. We have now reorganized some sections to explain and justify the clear logical flow in each step of the result section to build on the previous one in a clear scientific progression. Cancer relevance analysis of SIRT7 (Co-localization, expression, and cancer hallmarks) → Network analysis to identify how important SIRT7 is within its partners → GO and pathway analysis to provide mechanistic insights → Analysis of Context-dependent role of SIRT7 (Gene expression, patient survival, mutational analysis, immune association, and regulations), where we have addressed both similar and contradictory findings compared to literature → Therapeutic opportunity (Identified 3 potent novel inhibitors based on molecular docking, molecular dynamics and post-dynamics analysis). We hope this organized flow clearly justifies our pan-cancer study. |
| 2. | Background / Function of SIRT7 under Normal Conditions  The manuscript currently lacks sufficient description of SIRT7’s role in normal (non-cancer) biology. Please include a section that outlines the normal function(s) and mode(s) of action of SIRT7, with appropriate references. | Thank you for this suggestion. We have now presented a new paragraph in the background section of the introduction to address the normal (non-cancer) role of SIRT7 with appropriate references. |
| 3. | Gene vs Protein Nomenclature  Ensure that gene names are in italics (e.g. SIRT7) while protein names are in non-italics. This must be consistent throughout the manuscript (title, abstract, introduction, results, discussion). | We have not clearly addressed the gene name (Italic) and protein name (non-italic) and maintained them throughout the manuscript. |
| 4. | References & Citation Style  Use the PLOS ONE reference style | We have now maintained the PLOS ONE reference style throughout the manuscript. |
| 5. | Unreferenced Statements in Introduction  For example, the sentence:  “SIRT7 is considered an oncogene, as evidenced by Hepatocellular carcinoma (HCC), where the overexpression of SIRT7 in human HCC samples increased with tumor grade.”  This statement needs a proper citation. Please add references that support this assertion. | Thank you for this observation. We have now added a proper citation for this claim in the 3^rd^ paragraph of the introduction section. |
| 6. | Choice and Logic of Interaction Tools  You used multiple tools/databases to identify interacting partners (GeneMANIA, STRING, BioGRID). Please explain clearly:  o Why you selected more than one tool.  o What the principle/algorithm behind each tool is.  o What type of interactions each tool reveals (e.g. physical binding, genetic interaction, co-expression). | We have selected more than one tool because each tool serves a specific purpose. In the case of GeneMANIA, which utilizes a heuristic algorithm to integrate multiple functional association networks and predict gene function from a single process-specific network using label propagation [9], we have utilized it specifically to identify physical interactions and co-expression. Additionally, using STRING, which uses an average linkage algorithm for clustering to compute a combined score by combining the probabilities from the different evidence channels and correcting for the probability of randomly observing an interaction [10], we have specifically focused on protein-protein interaction because the main focus of STRING is to evaluate experimentally validated and predicted protein-protein interactions. Finally, we have used BioGRID for Gene Ontology terms analysis, which has no dedicated algorithm. It utilized manually curated data of Gene Ontology (GO) terms. Therefore, we have used BioGRID as it comprehensively categorizes GO terms based on query genes.  We have now clearly addressed them in 2.2.1. section of the manuscript. |
| 7. | Methods / Datasets  The methodology section needs clearer description of all datasets used, including origin, versions, preprocessing steps. For section 2.2.4 (“Automated Central Hub Identification”), describe in detail how central hubs are defined, the metrics used, and how thresholds were determined. | We have now clearly defined all datasets used, including origin, versions, and preprocessing steps.  For section 2.2.4 (“Automated Central Hub Identification”), we have now described in detail how central hubs are defined, the metrics used, and how thresholds were determined.  Now this section is presented in section 2.2.3. |
| 8. | Choice of Tools for Expression vs Survival Analysis  In section 2.3.1, you use TIMER2 for gene expression analysis and GEPIA2 for survival analysis. Explain why different tools were used for these related analyses. Could using GEPIA2 for both provide consistency? Clarify the rationale. | Our intention was to use the TIMER2 database because we have utilized this for the gene expression, mutational impact, and immune associations analysis in this study. However, we did not find any dedicated survival analysis tool in the TIMER2 database, and therefore, we have utilized GEPIA2 for survival analysis.  Both TIMER2 gene expression analysis and GEPIA2 survival analysis use the TCGA datasets, which ensures consistency. However, if you think adding gene expression data from GEPIA2 could be more valuable, we will definitely consider your suggestion. |
| 9. | Results vs Discussion Balance  In subsection 3.1.1, the manuscript presents:  “Fig 2. represents the structural quality assessment of the SIRT7 3D structure.”  But then there is no further discussion of what the findings mean. Please interpret figure results: what metrics in Fig 2 indicate good vs poor structure quality? What impact does this have for your downstream analysis? | We have now clearly interpreted **Figure 2** by mentioning what metrics indicate good and poor structure.  Our model demonstrates excellent local and global quality, as ERRAT 97.24 and Verify3D 86.75% meet or exceed recommended thresholds (≥95% and >80%), indicating strong atom-level and sequence‑to‑structure compatibility. Additionally, PROCHECK shows 90.5% of residues in most‑favored regions (threshold: ≥90%), reflecting high stereochemical quality. Finally, QMEAN −0.32 (0 is optimal) and a Z‑score of −9.06 (place the model within the X-ray crystallographic structural quality range), supporting its suitability for subsequent molecular docking and dynamics simulation studies. All the metric information that determines a good or poor quality structure has been provided by the tools or servers themselves.  These metrics help us to assess the quality of the SIRT7 3D structure that is required for subsequent downstream analysis of binding sites, molecular docking, and dynamics simulation.  We have now clearly mentioned them in section 3.1.1. |
| 10. | Figure Legends & Tool Attribution  All figure legends must clearly state which tool / software / database was used to generate the figure. Specifically for Figure 6, 7, 8: you must indicate in each legend whether the figure was produced using STRING, GeneMANIA, etc. | We have now clearly mentioned the tool/software/database with versions (if available) for all the figures in the figure legends. |
| 11. | Deep Neural Network Section Needs More Detail  The section 3.2.4 “Deep Neural Network Guided Central Hubs” is missing critical methodological details. Please include:  o What datasets were used.  o What features (independent variables) and labels/outcomes (dependent variables) were used.  o How the model was trained, validated, tested.  o Performance metrics.  o If possible, share the code (e.g. via GitHub) so that your analyses can be replicated. | We have now provided methodological details regarding the dataset, features, model training, validation, testing, and performance metrics.   1. The datasets are presented in **Supplementary Table S1**. We have now clearly mentioned that to develop our GCNConv model, we used target and network datasets generated using STRING 12. 2. Features are now clearly mentioned in **section 3.2.2 (Previously 3.2.4)**. The independent variables include the degree centrality, betweenness centrality, closeness centrality, clustering coefficient, eccentricity, average combined score, and stress, whereas the dependent variable is the predicted Score to rank SIRT7 among its interacting partners. 3. The model's performance was rigorously evaluated using a 60:20:20 train-validation-test set separation. This is mentioned in the **2.2.2 section.** 4. We have provided detailed performance metrics of the model: training, validation, and testing R2, MSE, and RMSE (**Table 1**), along with the training and validation Loss over 1000 epochs (**Figure 6**). 5. We have shared the code that is required to replicate or reproduce our model. The code is accessible at <https://github.com/tanjida255/SIRT7Net.git>. |
| Thank you for your additional feedback to revise our manuscript, which substantially improved the clarity, rigor, and overall quality of our work. | | |

**References**

1.	Aljada A, Saleh AM, Alkathiri M, Shamsa HB, Al-Bawab A, Nasr A. Altered sirtuin 7 expression is associated with early stage breast cancer. Breast Cancer (Auckl). 2015;9: 3–8. doi:10.4137/BCBCR.S23156;SUBPAGE:STRING:FULL

2.	Huo Q, Chen S, Zhuang J, Quan C, Wang Y, Xie N. SIRT7 Downregulation Promotes Breast Cancer Metastasis Via LAP2α-Induced Chromosomal Instability. Int J Biol Sci. 2023;19: 1528–1542. doi:10.7150/IJBS.75340

3.	Tsai YC, Greco TM, Cristea IM. Sirtuin 7 plays a role in ribosome biogenesis and protein synthesis. Molecular and Cellular Proteomics. 2014;13: 73–83. doi:10.1074/mcp.m113.031377

4.	Paredes S, Villanova L, Chua KF. Molecular pathways: Emerging roles of mammalian Sirtuin SIRT7 in cancer. Clinical Cancer Research. 2014;20: 1741–1746. doi:10.1158/1078-0432.CCR-13-1547/85857/AM/MOLECULAR-PATHWAYS-EMERGING-ROLES-OF-MAMMALIAN

5.	Simonet NG, Thackray JK, Vazquez BN, Ianni A, Espinosa-Alcantud M, Morales-Sanfrutos J, et al. SirT7 auto-ADP-ribosylation regulates glucose starvation response through mH2A1. Sci Adv. 2020;6. doi:10.1126/SCIADV.AAZ2590

6.	Shen H, Qi X, Hu Y, Wang Y, Zhang J, Liu Z, et al. Targeting sirtuins for cancer therapy: epigenetics modifications and beyond. Theranostics. 2024;14: 6726. doi:10.7150/THNO.100667

7.	Li S, Wu B, Zheng W. Cyclic tripeptide-based potent human SIRT7 inhibitors. Bioorg Med Chem Lett. 2019;29: 461–465. doi:10.1016/J.BMCL.2018.12.023

8.	Wei W, Jing ZX, Ke Z, Yi P. Sirtuin 7 plays an oncogenic role in human osteosarcoma via downregulating CDC4 expression. Am J Cancer Res. 2017;7: 1788. Available: https://pmc.ncbi.nlm.nih.gov/articles/PMC5622216/

9.	Mostafavi S, Ray D, Warde-Farley D, Grouios C, Morris Q. GeneMANIA: a real-time multiple association network integration algorithm for predicting gene function. Genome Biology 2008 9:1. 2008;9: 1–15. doi:10.1186/GB-2008-9-S1-S4

10.	von Mering C, Jensen LJ, Snel B, Hooper SD, Krupp M, Foglierini M, et al. STRING: known and predicted protein-protein associations, integrated and transferred across organisms. Nucleic Acids Res. 2005;33. doi:10.1093/NAR/GKI005
