# Supplementary material for: SIRT7 as a context-dependent biomarker and therapeutic target: Insights from a pan-cancer study
Source: PLoS One. 2026 Feb 5;21(2):e0342269. doi: 10.1371/journal.pone.0342269 (PMC12875470; doi:10.1371/journal.pone.0342269)
Supplement: Attachment — Submitted filename: Response to Reviewers.docx [file pone.0342269.s013.docx]

27 December, 2025

Firoz Ahmed
Academic Editor
PLOS One

Dear Editor,

Special gratitude to you for allowing us to resubmit a more revised and updated version of our manuscript entitled **"SIRT7 as a Context-Dependent Biomarker and Therapeutic Target: Insights from a Pan-Cancer Study" (Manuscript ID: PONE-D-25-42611R1)** to the PLOS ONE journal as an original research article. We have organized the manuscript based on the editor’s and reviewers’ comments. In response to valuable suggestions, we have modified, revised, organized, corrected, and narrowed the manuscripts. We have appropriately addressed all queries of Reviewer 2 in the response letter, line by line. We hope our response below will successfully address the reviewer’s specific points and improve the manuscript. Please let us know if you have any additional questions or comments. Finally, we would like to express our thanks and gratitude for your kind cooperation.

Sincerely yours,

Shahin Mahmud

Assistant Professor

Department of Biotechnology and Genetic Engineering

Mawlana Bhashani Science and Technology University, Santosh, Tangail-1902, Bangladesh.

Email: [shahin018mbstu@gmail.com](mailto:shahin018mbstu@gmail.com)

**Responses to the honorable reviewers’ s comments**

**Honorable Reviewer 2:**

| **SL.** | **Comments/Questions/Instructions/Guidelines** | **Responses of the Authors** |
| --- | --- | --- |
| 1. | While responding to reviewers, please highlight the added text in the revised manuscript and clearly mention the highlighted section and paragraph where the revisions have been made. It is also advisable to include page numbers and line numbers in the revised manuscript and refer to them accordingly in your responses. | Thank you for your valuable advice. We have added page numbers and line numbers in our manuscript. Also, in future submissions, we will adhere to your advice. |
| 2. | In response to my earlier comment, “The crystal structure of SIRT7 is already available ......”, you stated: “We have performed molecular docking targeting both the Electron Microscopic structure and the modeled structure with the experimentally validated SIRT7 inhibitor (PubChem CID: 155513088).” However, I do not find any docking results for the EM structure in the revised manuscript. Furthermore, selecting a cyclic tripeptide as the reference inhibitor for docking against small-molecule compounds is not rational, given that several small-molecule chemical inhibitors of SIRT7 are already reported. Please consult the following studies and perform a comprehensive literature review:  <https://www.sciencedirect.com/science/article/abs/pii/S1093326325002360>  <https://www.frontiersin.org/journals/cell-and-developmental-biology/articles/10.3389/fcell.2021.813233/full>  <https://www.sciencedirect.com/science/article/abs/pii/S0006291X1832552X> | Thank you for this important concern. We did not included the docking of EM structure earlier due to the superior performance of our modeled structure. However, we have included the docking results of all pharmacophore-based screened compounds for both the modeled structure and EM structure in the **Supplementary Table S4 Column 4**.  Again, thank you for your suggestion of this relevant literature. In our previous Supplementary Table S2 (Currently Supplementary Table S4), we already provided 2 experimentally validated SIRT7 inhibitors. One is based on the third literature you have suggested (<https://www.sciencedirect.com/science/article/abs/pii/S0006291X1832552X>). The control inhibitor we have used was named [SIRT7 inhibitor 97491](https://pubchem.ncbi.nlm.nih.gov/compound/146018038) (PubChem CID: 146018038). Due to a lower performance than the cyclic tripeptide ligand, we did not highlight it earlier. In the revised **Supplementary Table** **S2**, we have highlighted both the control inhibitors in Pink Color at the end of the Table. We have prioritized the cyclic tripeptide SIRT7 Inhibitor due to its greater binding potential with SIRT7 than other SIRT7 inhibitors (PubChem CID: 146018038). Moreover, the inhibitory potential of the cyclic tripeptide SIRT7 inhibitor is highly potent in the previous cell-line assay study. Therefore, earlier we had chosen both but prioritized the cyclic tripeptide SIRT7 inhibitor later. |
| 3. | Your response to the comment “While identifying the active site residues .......” is not satisfactory. Relevant data and previously designed inhibitors are available. Please refer to the studies mentioned above and revise your response accordingly. | Thank you for the guidance and clarification. Previously, we thought you had suggested adding only experimental evidence of inhibitory sites. Now, we have added relevant references for active site residues in the manuscript: **Page: 28, Line: 11,** and in the discussion section: **Page: 45, Line: 2,** where we have compared the active sites with previous studies. |
| 4. | Please verify, based on the above studies, whether you are targeting the same binding site or a different one. In either case, you must discuss your results in the context of these previously published findings. | We have targeted the same inhibitory sites that the above studies have suggested. We have now discussed and compared the active sites in our discussion section on **Page: 45, Line: 2-5**.  The 1^st^ study you have suggested (<https://www.sciencedirect.com/science/article/abs/pii/S1093326325002360>) used the catalytic residues: 169-277, which aligned with our study (**Figure 8A**).    **Figure 8 (A).** Active site of SIRT7.  The 2^nd^ study you have suggested (<https://www.frontiersin.org/journals/cell-and-developmental-biology/articles/10.3389/fcell.2021.813233/full>) utilized the almost exactly same binding site of our study: **PRO117**, **ASP118**, **ARG120**, **ASN168**, ASP170, **HIS187**, and LEU274.    **Figure.** The binding site from the previous study.  **Table 4.** Molecular docking analysis to capture interacting amino acids with compounds in the most suitable binding pose.    3^rd^ study (<https://www.frontiersin.org/journals/cell-and-developmental-biology/articles/10.3389/fcell.2021.813233/full>) did not provide the binding site information; instead presented the IC_50_ of [SIRT7 inhibitor 97491](https://pubchem.ncbi.nlm.nih.gov/compound/146018038) (PubChem CID: 146018038) = 0.325 μM.  We have now discussed and compared the findings with previous studies in the discussion section: **Page: 45, Line: 2-5.** |
| Thank you for your invaluable guidelines and advice, which are really necessary to significantly strengthen our manuscript. | | |

**Additional Editor Comments:**

| **SL.** | **Comments/Questions/Instructions/Guidelines** | **Responses of the Authors** |
| --- | --- | --- |
| 1. | Dear Dr. Mahmud,  Thanks for the submitting the revised manuscript and addressing the comments. Please address the comments of Reviewer #2. Please make sure the length of manuscript adhere to the journal guidelines. I found that the manuscript is still very long with 15 Figures and 7 Tables. | To reduce the length of the manuscript, we have presented some of the Figures and Tables in the Supplementary Section. Only the key Figures and Tables have now been retained in the main manuscript. All the Figures of the Manuscript have been provided in a minimum of 300 dpi in TIFF format in a zip file. |
